# Dietary Flavonoid Intake Is Inversely Associated with Cardiovascular Disease Risk as Assessed by Body Mass Index and Waist Circumference among Adults in the United States

**DOI:** 10.3390/nu9080827

**Published:** 2017-08-02

**Authors:** Rhonda S. Sebastian, Cecilia Wilkinson Enns, Joseph D. Goldman, Alanna J. Moshfegh

**Affiliations:** USDA, Agricultural Research Service, Food Surveys Research Group, 10300 Baltimore Avenue, BARC-West, Bldg 005, Rm 102, Beltsville, MD 20705-2350, USA; Cecilia.Enns@ars.usda.gov (C.W.E.); Joseph.Goldman@ars.usda.gov (J.D.G.); Alanna.Moshfegh@ars.usda.gov (A.J.M.)

**Keywords:** flavonoids, diet, cardiovascular disease risk, anthropometric measures, What We Eat in America, National Health and Nutrition Examination Survey (NHANES)

## Abstract

Although flavonoids may confer anti-inflammatory and anti-oxidant benefits, no research has examined if flavonoid intake is related to cardiovascular disease (CVD) risk defined by anthropometric measures in the USA population. This study sought to determine whether flavonoid intake is associated with combined body mass index (BMI) and waist circumference (WC) measures indicative of high, very high, or extremely high (“high+”) risk for CVD, using one day of 24-h recall data from adult (≥20 years) participants in What We Eat in America, National Health and Nutrition Examination Survey 2007–2010. Individuals were divided into categories of intake of total flavonoids and each flavonoid class, and adjusted estimates of the percentages at high+ CVD risk (based on BMI and WC, as per National Heart, Lung, and Blood Institute guidelines) were calculated. Inverse linear trends were found in percentages of adults at high+ CVD risk by intake of total flavonoids, anthocyanidins, flavan-3-ols, and flavanones (*p* < 0.01). For individuals in the highest (versus the lowest) intake category of anthocyanidins, flavan-3-ols, and flavanones, relative risk and confidence intervals (RR and CI, respectively) were 0.86 (99% CI: 0.79, 0.93), 0.88 (99% CI: 0.79, 0.98), and 0.89 (99% CI: 0.80, 0.98), respectively. Research is needed to determine whether the inverse relationships found in this study are applicable to CVD endpoints at the population level.

## 1. Introduction

Obesity is a major health concern both in the United States and abroad. In 2011–2014, 36% of USA adults were obese (body mass index (BMI) ≥ 30 kg/m^2^), with higher percentages among women, middle-aged and older adults, non-Hispanic (NH) blacks, and Hispanics, according to the Centers for Disease Control and Prevention, National Center for Health Statistics [1]. Both obesity and excess abdominal fat (a proxy for visceral fat, i.e., the fat surrounding internal organs) are well-documented risk factors for cardiovascular disease (CVD) and other chronic diseases, as well as for overall and disease-specific mortality [2,3,4,5]. Consequently, for nearly two decades, measurement of both BMI and waist circumference (WC; a surrogate marker of abdominal fat) has been an integral part of clinical guidelines for the assessment of overweight and obesity in USA adults [4,6]. Those guidelines contain a table of categories of risk for developing comorbidities including type 2 diabetes, hypertension, and/or CVD (hereafter termed “CVD risk”, since diabetes and hypertension both increase risk for CVD) that are based on both BMI and WC together [4,6]. For individuals with a BMI between 25 and 34.9 kg/m^2^ (54% of USA adults in 2007–2010) [7], WC improves the predictive power of disease risk classification over and above BMI [4].

Extensive research has been conducted to identify dietary components (beyond the consumption of excess energy) that may influence the accumulation of excess body fat. Among such dietary components are flavonoids, which are a large, diverse group of bioactive polyphenolic compounds found in plants. The six main classes of flavonoids (with examples of foods that provide them to adults in the USA) are the following: Anthocyanidins (fruits, especially berries), flavan-3-ols (tea), flavanones (citrus fruits and juices), flavones (tea, peppers, and celery), flavonols (tea, onions, and potatoes), and isoflavones (soy products) [8]. Mechanistic studies have suggested various plausible means by which flavonoids (or flavonoid-containing foods and beverages) may impact body fatness, such as by decreasing energy intake [9,10], increasing energy expenditure and fat oxidation [11], influencing macronutrient absorption and uptake [12], and inhibiting adipogenesis [13].

Although a number of studies have identified favorable associations between flavonoids (or flavonoid-rich foods/beverages/supplements) and BMI and/or WC [11,14,15,16,17,18,19,20], to our knowledge no study has been conducted to determine the relationship between flavonoid intake and CVD risk when that risk is assessed on the basis of BMI and WC together. Further, the majority of the studies that have looked at these anthropometric measures individually in relation to flavonoid intake have not analyzed dietary data from nationally representative populations. Therefore, the purpose of this study was to examine associations between intake of flavonoids (total and five classes) and CVD risk as assessed by BMI and WC jointly among adults age ≥20 years overall and by sex (males, females) via analysis of a sample representative of the United States population.

## 2. Methods

### 2.1. Data Source and Sample

Data used in this study were collected between 2007 and 2010 in What We Eat in America (WWEIA), the dietary intake component of the National Health and Nutrition Examination Survey (NHANES) [21,22]. The NHANES sample was constructed to be representative of the civilian, noninstitutionalized USA population [23]. A complex, multistage, area probability sample design was used to select persons within households. During 2007–2010, NH blacks, Hispanics, people age ≥80 years, and low income persons were oversampled to improve the accuracy of estimates for these population groups. Among adults age ≥20 years, the examination response rate (i.e., the percentage of individuals selected into the sample who were interviewed in the Mobile Examination Center (MEC)) was 70.6% in 2007–2008 and 72.2% in 2009–2010 [24].

Because the BMI and WC standards used in assessing CVD risk apply to adults, this analysis was restricted to individuals age ≥20 years. During their visit to the MEC, 11,182 adults provided an in-person 24-h dietary recall, the intake used in this analysis. Excluded were those who were pregnant and/or lactating (*n* = 188) or were missing weight, height, and/or WC data (456 total: 219 males, 237 females). The final analytic sample included 10,538 adults (5232 males and 5306 females). Written informed consent was obtained from all participants.

The survey protocol was approved by the National Center for Health Statistics Research Ethics Review Board. The current study was a secondary analysis and thus exempt from further review under Title 45 Code of Federal Regulations section 46.101(b).

### 2.2. Assessment of Flavonoid Intakes

Intakes in this study are based on foods and beverages, but not supplements, reported in WWEIA, NHANES. The data were collected by trained interviewers fluent in English and Spanish using the United States Department of Agriculture (USDA) Automated Multiple-Pass Method (AMPM) for the 24-h recall [25]. Question sequences in the AMPM were crafted to elicit sufficient detail to assign the most appropriate USDA food code from the USDA Food and Nutrient Database for Dietary Studies (FNDDS) [26,27]. Neither the AMPM nor the FNDDS was specifically designed for the analysis of flavonoid intakes. However, the AMPM’s detailed, food-specific question sequences include many questions about factors that permit discrimination between food codes differing in flavonoid content. For example, a participant who reports consuming “beans” is asked what variety of beans was consumed, which permits assignment of food codes that differ in flavonoid content (e.g., kidney beans versus pinto beans versus black beans). The AMPM collects information about foods and beverages as consumed, resulting in comprehensive estimation of the intake of flavonoid-rich foods that are typically ingredients in mixtures—such as onions, potatoes, celery, and peppers. 

Versions of the FNDDS appropriate to each survey cycle were applied in assigning USDA food codes; version 4.1 was used for 2007–2008, and version 5.0 was used for 2009–2010 [27]. The USDA food code also served as the link between the WWEIA, NHANES foods/beverages and the Database of Flavonoid Values for USDA Food Codes 2007–2010 (hereafter referred to as the “Flavonoid Database”) [28], which provides values for 29 individual flavonoids, six flavonoid classes, and total flavonoids for all food codes (*n* = 7273) used in processing data in WWEIA, NHANES 2007–2010 [29]. Application of the Flavonoid Database to the dietary intake data yielded the Flavonoid Intake Data Files from WWEIA, NHANES 2007–2010 [28], which allow calculation of flavonoid intake estimates representative of the USA population.

Aggregate daily intakes of total flavonoids (the sum of the 29 individual flavonoids) and five of the flavonoid classes (anthocyanidins, flavan-3-ols, flavanones, flavones, and flavonols) from all foods and beverages were estimated. In addition to catechins, the flavan-3-ol class includes derived tannins (theaflavins and thearubigins) [29], but it excludes condensed tannins (proanthocyanidins) because sufficient data are not yet available for the full range of survey foods and beverages. Although included in total flavonoids, isoflavones were not analyzed separately because: (a) intakes in the USA are so low [8] that no biological effect would be expected [30]; and (b) intakes derived using the Flavonoid Database are underestimated, due to the general exclusion of isoflavone contributions from functional ingredients such as soy additives that serve as stabilizers and emulsifiers [29].

In order to assess contributions by food groups to flavonoid intakes (total and class), each food or beverage reported was classified into one of 76 mutually exclusive groups, using the appropriate versions of the WWEIA Food Categories [31] (see the “Files” tab in that reference) as a foundation.

### 2.3. Anthropometric Measures

On the day the in-person dietary recall was conducted, trained health technicians measured weight, height, and WC in the MEC using standardized protocols and procedures, including electronic data capture [32,33]. Body weight was measured with the participant wearing standard MEC examination clothing, and height was measured using a stadiometer. The WC measurement was taken at the top of the iliac crest, as described in the clinical guidelines [4]. 

### 2.4. Assessment of CVD Risk

As was done in the National Institutes of Health (NIH) National Heart, Lung, and Blood Institute *Practical Guide* [4] (see Table 2 on p. 10 of that report), categories of CVD risk in the present study are based on BMI and, for those with BMI 25–34.9 kg/m^2^, sex-specific WC cutoffs (>102 cm for men, >88 cm for women). WC does not affect risk classification among individuals with BMI ≥ 35.0 kg/m^2^. This study sought to determine flavonoid associations with any combination of BMI and WC leading to classification as being at “high”, “very high”, or “extremely high” risk (hereafter, termed “high+ CVD risk”).

### 2.5. Definitions of Adjustment Variables/Covariates

The analysis included a number of sociodemographic and lifestyle characteristics (all self-reported except sex) and dietary intake components as adjustment variables when assessing the relationship between CVD risk and flavonoid intake. Sociodemographic characteristics included age, sex, race/ethnicity, and family income as a percentage of poverty (also termed Poverty Income Ratio (PIR)).

Lifestyle characteristics included exercise status, smoking status, and health status. Exercise status was calculated as minutes of recreational physical activity per week, with each vigorous-intensity minute counted as two moderate-intensity minutes [34]. Smoking status was based upon whether participants reported having smoked 100 cigarettes in their lives (no = never smoker) and, if yes, whether they smoked at the time of the survey (no = former smoker, yes = current smoker). Health status was self-reported as excellent, very good, good, fair, or poor.

Dietary intake components associated with BMI and/or WC—energy, dietary fiber, and added sugars—were also included in the model. (Solid fats and refined grains were also examined but did not explain considerable variance over and above that accounted for by the other dietary components and, therefore, were omitted from the model.) The appropriate versions of the FNDDS were used to calculate intakes of energy and dietary fiber [27], and those of the USDA Food Patterns Equivalents Database to calculate intakes of added sugars [35].

### 2.6. Statistical Analyses

Analyses were performed using SAS©-callable SUDAAN, release 11.0 (2012; RTI International, Research Triangle Park, NC, USA), adjusting for survey design effects resulting from NHANES’ complex, multistage probability sampling [23]. Application of dietary sample weights yielded estimates representative of the USA adult population.

For total flavonoids and each flavonoid class separately, respondents were divided into four categories of intake. Estimates of anthocyanidin and flavanone intake were zero for a sizable portion of the population (34% and 35%, respectively, of all adults). For these two flavonoid classes, all individuals with zero intake were assigned to the first category, and those with non-zero intake were divided into tertiles. Dividing respondents into quartiles per se was appropriate for total flavonoids, flavan-3-ols, flavones, and flavonols, because 10% or fewer adults had zero intakes.

Adjustment variables were examined by quartile of total flavonoid intake. Significant differences among means were identified via χ^2^ tests of independence for the (categorical) demographic and lifestyle characteristics and via analysis of variance (ANOVA) for the (continuous) dietary intake components.

Logistic regression was employed to ascertain the likelihood of high+ CVD risk by flavonoid (total and class) intake and the associated risk ratios. Adjustment variables were added sequentially in three steps: first, energy, sex, and age; next, race/ethnicity, income, and all lifestyle characteristics; and finally, in the fully fitted model, dietary fiber and added sugars. These model-adjusted prevalence estimates and model-adjusted risk ratios were calculated using the average marginal prediction approach described by Graubard and Korn [36] and Bieler et al. [37], which is designed to permit unbiased comparisons of risk between population subgroups after controlling for differences in their covariate distributions. Orthogonal polynomial contrasts were examined to identify linear trends in the prevalence of individuals at high+ CVD risk by flavonoid intake category. Model-adjusted risk ratios comparing the prevalence estimates of high+ CVD risk in a given category of flavonoid intake relative to that of the lowest (referent) category were computed with their corresponding 99% confidence intervals. Since obesity rates differ significantly by sex [1], it was hypothesized that the percentages at high+ CVD risk would also differ by sex. Thus, the analysis was repeated, applying the fully adjusted model only, and stratified by sex, using sex-specific flavonoid intake ranges for categorization.

For all analyses, *p*-values < 0.01 were considered statistically significant.

## 3. Results

In 2007–2010, for all adults age ≥20 years, mean (median) total flavonoid intake in the first quartile was 9.3 (9.3) mg; second, 33.4 (32.0) mg; third, 104.3 (89.6) mg; and fourth, 839.4 (603.0) mg. Demographic and lifestyle characteristics and dietary intake components are shown in Table 1. All variables examined differed significantly (*p* < 0.01) across quartiles of total flavonoid intake. Individuals with higher total flavonoid intake were more likely to be older (≥40 years), NH white, higher income, more active, and never or former smokers and to assess their health as excellent than were those with lower flavonoid intakes. As determined by orthogonal polynomial contrasts, significant positive linear trends were found in intakes of energy and dietary fiber with increasing quartile of total flavonoids intake.

Food/beverage groups contributing 5% or more of the total intake of flavonoids (total and class) are shown in Table 2. A single beverage group, tea, provided over three-fourths of total flavonoids intake (78% of intake for all adults), and nearly all of flavan-3-ols intake (94%). Tea was also a major contributor to flavones (10%) and flavonols (37%). Four groups of berries (blueberry, cranberry, strawberry, and other), plus the berries included as ingredients in yogurt, were the primary contributors to anthocyanidin intake, as were citrus fruits and juices to flavanone intake and soy products (including protein powders and milk substitutes) to isoflavone intake. Because flavones and flavonols are found in vegetables that are ubiquitous in mixtures (e.g., onions, potatoes, celery, peppers, and parsley), mixed dishes were among the top contributors to these flavonoid classes. Generally, though not uniformly, top sources were comparable for males and females.

As shown in Table 3, in 2007–2010, 31.2% of all adults were of normal weight or underweight (BMI < 25.0) and thus classified as being at average CVD risk, with significantly (*p* < 0.01) fewer males than females in this risk class (27.1% vs. 35.2%). However, more overweight (BMI 25.0–29.9) males than females had normal WC (26.3% vs. 5.8%) and were thus assessed as being at only increased risk rather than high risk. Over one-half (53.0%) of all adults in the USA were at high+ CVD risk. A higher percentage of females than males was classified as being at high+ CVD risk, due to higher percentages of females in the high risk (specifically, overweight with large WC) and extremely high risk categories.

For all adults age ≥20 years, the likelihood of being classified at high+ CVD risk (assessed on the basis of BMI and WC) by category of flavonoid intake is shown in Table 4. After adjustment for all demographic and lifestyle characteristics and dietary intake components (Model 3), significant (*p* < 0.01) inverse linear trends were found in prevalence of high+ CVD risk by intake of total flavonoids, anthocyanidins, flavan-3-ols, and flavanones. Adults in quartile 3 of total flavonoid intake were 13% less likely to be classified at high+ CVD risk than were those in quartile 1, the referent category. The relative risk (RR) of high+ CVD risk was 8% to 14% lower among adults in categories 3 and 4 of anthocyanidin intake; 12% to 14% among those in categories 2, 3, and 4 of flavan-3-ol intake; and 11% among those in category 4 of flavanone intake. An inverse linear trend in prevalence of being classified at high+ CVD risk by quartile of flavone intake was observed after adjustment for energy, sex, and age, but that finding was attenuated after race/ethnicity and lifestyle characteristics were included in the model. No linear trend in prevalence of high+ CVD risk by flavonol intake was noted.

In analyses stratified by sex and fully adjusted (Table 5), inverse linear trends were observed in prevalence of being classified at high+ CVD risk by category of intake for total flavonoids (males only), anthocyanidins (males and females), and flavan-3-ols (males only). Males in the second, third, and fourth quartiles of total flavonoids intake were 14% to 20% less likely to be classified at high+ CVD risk than were those in the lowest quartile. The RR of high+ CVD risk was 16% lower among females in the highest category of anthocyanidin intake, and 17% to 24% among males in categories 2 to 4 of flavan-3-ol intake. No linear trends were found in prevalence of being classified at high+ CVD risk among males or females by intake of flavanones, flavones, or flavonols.

## 4. Discussion

In this study, relationships were identified between flavonoid intake and prevalence of being classified at high+ CVD risk based on BMI and WC in a large, nationally representative sample of USA adults. To our knowledge, this is the first study to examine the relationship between flavonoid intake and CVD risk using BMI and WC together as a marker of risk status for type 2 diabetes, hypertension, and CVD.

Intakes of total flavonoids, anthocyanidins, flavan-3-ols, and flavanones were inversely associated with high+ CVD risk, whereas intakes of flavones and flavonols were not. When stratified by sex, the inverse linear relationship between anthocyanidin intake and high+ CVD risk was maintained for both sexes, but the inverse associations for total flavonoids and flavan-3-ols were seen among males only, and no associations were seen for flavanones.

It is challenging to integrate these novel findings with the existing literature. All previous research that investigated BMI and/or WC in relation to flavonoid intake assessed these measures individually. One study that is more comparable than most was conducted on NHANES 2007–2012 data [14]. Using a proprietary flavonoid database and analyzing multivariate-adjusted models to determine associations between flavonoid intake and CVD risk factors that included BMI and WC, Kim et al. found an inverse linear trend between anthocyanidin intake and BMI, but no relationships between other flavonoid classes and BMI or between any flavonoid class and WC. Other research reporting associations between flavonoid intake and BMI and/or WC is less comparable because of methodological differences. A few studies that investigated flavonoid intake in relation to WC (as a component of metabolic syndrome) differed from the present study not only in the anatomical landmark for measurement of WC but also in the cutpoints (mostly, more restrictive) for high WC [38,39]. Numerous other studies incorporating analysis of flavonoid intake in relation to these anthropometric measures analyzed BMI and WC only as baseline characteristics, and associations were based on unadjusted or minimally adjusted (e.g., energy-adjusted) data, so their findings of inverse relationships with flavonoid intake are not truly parallel to those presented here [40,41,42].

With flavan-3-ols accounting for about 80% of total flavonoid intake in the USA [28] (see “Data Tables” in that reference), it is unsurprising that associations noted between total flavonoids and high+ CVD risk are similar to those for flavan-3-ols. Due to much higher flavan-3-ol content in tea relative to other foods, tea was the single predominant source of flavan-3-ols. Much research has been conducted linking green tea catechins (monomeric flavan-3-ols) to more favorable anthropometric measures [11,17,19,20]. One might question the relevance of those findings to the current study, since most tea consumed in the USA is black tea [43], which is higher in flavan-3-ol dimers (theaflavins) and polymers (thearubigins) and much lower in catechins relative to green tea [44]. Nevertheless, our results are consistent with that work: Individuals in the highest quartile of flavan-3-ol intake, nearly all of whom consumed tea on the intake day, were significantly less likely to be at high+ CVD risk. Moreover, the 14% reduced RRs that were also observed for individuals in quartile 3 (of whom very few consumed tea) and quartile 2 (who consumed no tea), suggest that the observed association was not merely an effect of the caffeine contained in tea (or synergy between caffeine and tea-specific flavan-3-ols), as has been suggested [18].

Longitudinal research mostly supports our finding that body measures indicative of increased CVD risk are inversely associated with intake of anthocyanidins and flavanones and/or food sources of these flavonoid classes. In the Nurses’ Health Study, Health Professionals Follow-up Study, and Nurses’ Health Study II, inverse associations between intake of anthocyanidins and weight gain were found [45]. In the same cohorts and in the European Prospective Investigation into Cancer and Nutrition Study, negative weight change or decreases in WC were observed with increased intake of berries [46] and yogurt [16,47], foods that together accounted for over 40% of anthocyanidins in the present study. However, in the Women’s Health Study, both berries [48] and yogurt [49] were associated with increased likelihood of becoming overweight or obese over time. Likewise, consumption of citrus fruits, which are major contributors of flavanones, has been found to be protective against weight gain by some [48], despite other investigations failing to detect an association between flavanone intake and weight change [45]. No long-term investigations analyzing flavonoid associations with WC specifically were located, but a meta-analysis of prospective cohort studies concluded that higher intakes of fruits, but not vegetables, were associated with lower increases in WC over time [50]. 

Unlike anthocyanidins, neither flavone or flavonol intake has been found to be associated with long-term weight change [45]. Conversely, some of the foods identified in the present study as contributors to these flavonoid classes have been shown to be associated with increases in BMI and/or WC. Beer, shown in Table 2 to be one of the leading sources of flavonols (particularly among males), has been associated with increases in both weight [47] and WC [16,51]. In addition, the relationship between vegetables that are sources of both flavones and flavonols and weight change and WC is not uniform across specific vegetables. Potatoes, particularly fried potatoes, are associated with weight gain [46,47] and increases in WC [52], but inconsistent results have been reported with regard to other vegetable classes [46,48]. In addition, our results indicate that a substantial proportion of flavones and flavonols are obtained from vegetables that are ubiquitous in mixed dishes, e.g., onions and peppers. Other foods that are also common ingredients in mixed dishes, such as meats and white bread, have been shown to be associated with weight gain [47] and increases in WC [16] and, thus, may mask any potential beneficial associations between those flavonoid classes and BMI and WC. 

The categories of risk classification used in this study were crafted by experts based on the literature linking elevations in BMI and WC with increased risk of developing various chronic diseases [4]. Although the outcome variable in the present study was given the abbreviated description “high+ CVD risk”, it is, as indicated previously, an umbrella term that includes high, very high, and extremely high risk for not only CVD but also type 2 diabetes and hypertension [4]. Several prospective cohort studies have revealed inverse relationships between flavonoid intakes and incidence of various chronic diseases for which obesity, especially abdominal obesity, is a risk factor, e.g., heart disease [40,41,42,53], ischemic stroke [53,54], type 2 diabetes [55], and hypertension [39,56]. Our findings suggest that similar inverse associations between flavonoid intake and CVD endpoints may be found for the USA adult population as a whole. That possibility deserves further study.

This study has a number of strengths. First, findings are generalizable to the USA population as a whole due to the national representativeness of the NHANES sample, and thus are applicable to all race/ethnic, income, and education groups, including those underrepresented in many cohort studies investigating associations between flavonoid intake and health outcomes. Second, the 24-h dietary recall method used in this study, USDA’s AMPM, is highly regarded [57] and has been validated for energy and sodium intakes [25,58]. Third, the use of standardized methods and trained technicians to collect anthropometric data [32,33] enhanced the accuracy of those measurements relative to self reports. Finally, because the flavonoid composition of a wide range of foods and beverages was available, it was possible to comprehensively assess flavonoid intake from all dietary sources including mixed foods, items which made substantial contributions to several of the flavonoid classes. Failure to include these sources has been identified as a common component in the widespread underestimation of flavonoid intakes in research [59].

Some limitations are worth noting. First, the validity of using BMI and WC to assign risk has been called into question due to their inability to differentiate between visceral and subcutaneous adipose tissue [60], as well as to racial/ethnic differences in both relevant cut points [61,62] and risk prediction accuracy [62]. On the other hand, in addition to the research underlying the National Heart, Lung, and Blood Institute guidelines [4], large studies with clinical endpoints have reported superior classification of CVD risk when both BMI and WC are considered together [3,63]. Nevertheless, assessment of CVD risk on the basis of anthropometric measures alone may not accurately reflect cardiometabolic markers in some individuals [64], and could result in misclassification of risk category. Second, examination of associations between intakes of other flavonoid classes (e.g., proanthocyanidins) or other polyphenols and high+ CVD risk was impossible, because information on the content of these compounds is not yet available for the wide range of foods reported in NHANES 2007–2010. Third, underreporting is a known issue in dietary surveys [25]. However, 24-h recalls as used in the present study are often employed as the standard against which food frequency questionnaires (which are often used in flavonoids research) are evaluated to judge the completeness of their collection of flavonoids and other nutrients and dietary components [65,66,67]. Lastly, use of one day of dietary data could have resulted in some misclassification of individuals into categories of flavonoid intake, since a single 24-h recall is not necessarily reflective of usual intake. However, among the 9178 individuals in this study who provided two days of intake data, using the two-day average to assign individuals to quartiles of total flavonoid intake results in 62.4% of individuals being classified in the same category as their Day 1 classification and an additional 32.6% being classified into an adjacent category. Similar patterns were observed for the individual flavonoid classes, and for males and females separately. Thus, most individuals (95.0%) were classified similarly whether one or two 24-h recalls were employed to estimate flavonoid intake. 

In conclusion, intakes of total flavonoids, anthocyanidins, flavan-3-ols, and flavanones were inversely associated with prevalence of high+ CVD risk (as assessed by BMI and WC) in a large, nationally representative sample of USA adults. An extensive body of research identifies plant foods as important components of a cardioprotective diet. Findings from this study support that conclusion, particularly with regard to foods and beverages that are rich sources of these polyphenolic compounds. Research examining whether these same associations with flavonoid intake are applicable to CVD endpoints at the population level is needed. 

## Figures and Tables

**Table 1:** Characteristics by quartile of total flavonoid ^1^ intake, adults age ≥20 years, WWEIA, NHANES 2007–2010.

Characteristic	*n*	Quartile of Flavonoid Intake ^2^				*p* ^3^
		1	2	3	4	
All, *n*	10,538	2819	2693	2663	2363	
Demographic:						
Sex, %:						0.002
Male	5232	45.3	50.7	50.5	47.8	
Female ^4^	5306	54.7	49.3	49.5	52.2	
Age (year), %:						<0.001
20–39	3393	42.7	39.0	34.1	30.2	
40–59	3555	37.3	38.9	39.6	42.4	
60+	3590	20.0	22.0	26.3	27.4	
Race/ethnicity, %:						<0.001
NH white	5046	66.8	67.5	70.4	74.3	
NH black	2007	13.5	11.2	10.2	9.7	
Hispanic ^5^	3007	15.2	15.8	14.8	7.8	
Other ^6^	478	4.5	5.5	4.7	8.2	
Income as PIR ^7^, %:						<0.001
0–130	3037	26.4	19.8	16.8	16.3	
131–350	3681	36.0	32.6	30.7	30.6	
>350	2882	30.7	40.3	45.4	45.7	
Not reported	938	6.8	7.3	7.2	7.4	
Lifestyle:						
Exercise status ^8^, %:						<0.001
Inactive/low	7336	71.3	64.6	55.5	63.0	
Moderate	1134	10.0	10.7	15.4	13.7	
Active	2068	18.8	24.7	29.2	23.3	
Smoking status ^9^, %:						<0.001
Never smoker	5567	47.6	53.5	56.5	55.3	
Former smoker	2623	21.5	23.5	27.5	25.1	
Current smoker	2348	30.9	23.0	16.0	19.6	
Self-assessed health status, %:						<0.001
Excellent/very good	4101	40.6	49.3	52.9	50.1	
Good	3775	37.6	31.6	32.2	33.8	
Fair/poor	2662	21.9	19.1	14.9	16.0	
Dietary:						
Energy, kcal (SE)	10,538	1816 (24)	2207 (22)	2304 (27)	2225 ^10^ (39)	<0.001
Dietary fiber, g (SE)	10,538	11.0 (0.2)	16.4 (0.3)	20.5 (0.3)	18.3 ^10^ (0.6)	<0.001
Added sugars, g (SE)	10,538	77.2 (2.6)	77.8 (2.3)	69.0 (2.1)	84.0 (3.1)	<0.001

Abbreviations: WWEIA, What We Eat in America; NHANES, National Health and Nutrition Examination Survey; NH, non-Hispanic; PIR, Poverty Income Ratio; SE, standard error. ^1^ Total flavonoids = sum of 29 individual flavonoids in six classes; includes anthocyanidins, flavan-3-ols (catechins, theaflavins, and thearubigins), flavanones, flavones, flavonols, and isoflavones. ^2^ For intake ranges by quartile, see the last two tables in this article. ^3^ Data were analyzed using χ^2^ tests for the (categorical) demographic and lifestyle characteristics and analysis of variance for the (continuous) dietary intake components. ^4^ Excludes pregnant and lactating females. ^5^ Includes Mexican Americans and other Hispanics. ^6^ Includes both individuals who were of races other than those listed and those who were multiracial. ^7^ PIR is the ratio of family income to poverty (expressed as a percentage). ^8^ Includes only recreational activity, with each vigorous-intensity minute counted as the equivalent of two moderate-intensity minutes; levels correspond to those in the 2008 Physical Activity Guidelines for Americans [34]. ^9^ Participants who reported smoking <100 cigarettes in their lives were classified as “never smokers”, those who had smoked ≥100 cigarettes but did not smoke at all at the time of the survey were classified as “former smokers”, and those who smoked cigarettes every day or some days at the time of the survey were classified as “current smokers.” ^10^ Significant positive linear trend across quartiles of total flavonoid intake, as determined by (first order) orthogonal polynomial contrasts (*p* < 0.001).

**Table 2:** Food/beverage groups providing ≥5% of total intake ^1^ of flavonoids, adults age ≥20 years, for all and by sex, WWEIA, NHANES 2007–2010.

Flavonoid Class	Rank	Food/Beverage Group (% Contribution to Flavonoid Class Intake)		
		All	Males	Females
Total flavonoids	1	Tea (78)	Tea (78)	Tea (78)
Anthocyanidins	1	Blueberries (22)	Blueberries (19)	Blueberries (24)
	2	Wine (14)	Wine (16)	Wine (12)
	3	Grapes (10)	Grapes (12)	Strawberries (9)
	4	Strawberries (8)	Strawberries (7)	Grapes (9)
	5	Miscellaneous vegetables ^2^ (7)	Non-citrus juice other than apple, 100% (6)	Miscellaneous vegetables ^2^ (8)
	6	Cranberries (6)	Cranberries (5)	Yogurt (6)
	7	Yogurt (5)	Miscellaneous vegetables ^2^ (5)	Cranberries (6)
	8	Non-citrus juice other than apple, 100% (5)		Other berries (5)
Flavan-3-ols	1	Tea (94)	Tea (94)	Tea (94)
Flavanones	1	Orange juice, 100% (59)	Orange juice, 100% (63)	Orange juice, 100% (54)
	2	Oranges (19)	Oranges (19)	Oranges (21)
	3	Other citrus fruits (6)		Other citrus fruits (8)
	4			Fruit drinks, <100% juice (5)
Flavones	1	Mixed dishes (25)	Mixed dishes (26)	Mixed dishes (23)
	2	Sweet peppers (10)	Sweet peppers (11)	Tea (10)
	3	Tea (10)	Tea (9)	Sweet peppers (8)
	4	Miscellaneous vegetables ^2^ (7)	Hot peppers (8)	Miscellaneous vegetables ^2^ (7)
	5	Melons (6)	Miscellaneous vegetables ^2^ (7)	Lettuce and lettuce-based salads (6)
	6	Condiments and sauces, non-soy-based (6)	Condiments and sauces, non-soy-based (7)	Melons (6)
	7	Lettuce and lettuce-based salads (5)	Melons (6)	Parsley (6)
	8	Hot peppers (5)	Lettuce and lettuce-based salads (5)	Condiments and sauces, non-soy-based (5)
Flavonols	1	Tea (37)	Tea (35)	Tea (40)
	2	Mixed dishes (14)	Mixed dishes (15)	Mixed dishes (14)
	3	Beer (6)	Beer (9)	Apples (5)
	4	Onions (5)	Onions (5)	Onions (5)
Isoflavones	1	Protein powders (38)	Protein powders (50)	Milk substitutes (23)
	2	Milk substitutes (18)	Milk substitutes (13)	Protein powders (22)
	3	Processed soy products (16)	Processed soy products (11)	Processed soy products (22)
	4	Nutrition bars (8)	Nutrition bars (7)	Mixed dishes (11)
	5	Mixed dishes (8)	Beans, peas, legumes (7)	Nutrition bars (10)
	6	Beans, peas, legumes (5)	Mixed dishes (5)	

Abbreviations: WWEIA, What We Eat in America; NHANES, National Health and Nutrition Examination Survey. ^1^ For mean intakes of flavonoids by gender and age, race/ethnicity, and income, see Reference [28]. ^2^ The group “miscellaneous vegetables” includes vegetables that were not classified into the other, more specific groups in this analysis—namely, tomatoes, carrots, other red/orange vegetables, lettuce and lettuce-based salads, dark-green vegetables (excluding lettuce), potatoes (three groups: baked/boiled; French fries and other fried; and mashed and mixtures), corn and other starchy vegetables, onions, sweet peppers, hot peppers, celery, and parsley.

**Table 3:** Distribution of individuals by CVD risk class ^1^, adults age ≥20 years, for all and by sex, WWEIA, NHANES 2007–2010.

CVD Risk Class		Associated BMI and WC ^2^	All	Males	Females ^3^
			% (SE)		
Average		BMI < 25.0	31.2 (0.9)	27.1 ^a^ (1.3)	35.2 ^b^ (1.0)
Increased		BMI 25.0–29.9, normal WC	15.8 (0.5)	26.3 ^a^ (0.8)	5.8 ^b^ (0.4)
High+:			53.0 (1.0)	46.6 ^a^ (1.3)	59.1 ^b^ (1.1)
	High:	BMI 25.0–29.9, large WC	17.9 (0.7)	12.3 (0.6)	23.2 (1.1)
		BMI 30.0–34.9, normal WC	1.3 (0.2)	2.5 (0.3)	0.1 (#)
		Total	19.2 (0.8)	14.8 ^a^ (0.7)	23.3 ^b^ (1.1)
	Very high:	BMI 30.0–34.9, large WC	19.2 (0.5)	20.0 (0.8)	18.4 (0.7)
		BMI 35.0–39.9	8.9 (0.4)	7.6 (0.5)	10.1 (0.4)
		Total	28.1 (0.7)	27.6 (1.1)	28.5 (0.7)
	Extremely high	BMI ≥ 40.0	5.8 (0.2)	4.2 ^a^ (0.3)	7.3 ^b^ (0.4)

Abbreviations: CVD, cardiovascular disease; WWEIA, What We Eat in America; NHANES, National Health and Nutrition Examination Survey; BMI, body mass index; WC, waist circumference; SE, standard error; high+, incorporates high, very high, and extremely high CVD risk categories. # Indicates a non-zero value too small to report. ^a,b^ Within risk class, estimates with different superscript letters differ significantly (*p* < 0.01). In the high risk and very high risk classes, only estimates in the “total” row were tested for significant differences. ^1^ CVD risk class assigned on the basis of BMI and WC, as delineated in Reference [6]. ^2^ BMI is reported in kg/m^2^. Normal WC ≤ 102 cm for men and ≤88 cm for women; large WC > 102 cm for men and >88 cm for women. ^3^ Excludes pregnant and lactating females.

**Table 4:** RR ^1^ of being classified at high+ CVD risk ^2^ by category ^3^ of flavonoid intake, adults age ≥20 years, WWEIA, NHANES 2007–2010.

RR by Model, by Flavonoid Class	Category of Flavonoid Intake				*p* for Trend ^4^
	1	2	3	4	
Total flavonoids ^5^:					
Range of intake, mg	≤19.1	19.2–53.1	53.2–230.8	>230.8	
RR (99% CI):					
Model 1	1.00	0.90 (0.82, 0.99)	0.82 (0.74, 0.91)	0.85 (0.76, 0.96)	<0.001
Model 2	1.00	0.93 (0.85, 1.01)	0.85 (0.78, 0.93)	0.89 (0.80, 1.00)	0.003
Model 3	1.00	0.93 (0.86, 1.01)	0.87 (0.80, 0.95)	0.91 (0.82, 1.01)	0.009
Anthocyanidins:					
Range of intake, mg	0.0	0.1 ^6^–1.8	1.9–10.1	>10.1	
RR (99% CI):					
Model 1	1.00	0.92 (0.83, 1.02)	0.89 (0.82, 0.96)	0.80 (0.73, 0.89)	<0.001
Model 2	1.00	0.93 (0.84, 1.02)	0.90 (0.84, 0.96)	0.84 (0.77, 0.90)	<0.001
Model 3	1.00	0.93 (0.85, 1.03)	0.92 (0.85, 0.99)	0.86 (0.79, 0.93)	<0.001
Flavan-3-ols:					
Range of intake, mg	≤3.3	3.4–12.7	12.8–121.8	>121.8	
RR (99% CI):					
Model 1	1.00	0.85 (0.78, 0.93)	0.81 (0.72, 0.91)	0.84 (0.75, 0.94)	<0.001
Model 2	1.00	0.86 (0.79, 0.93)	0.84 (0.75, 0.95)	0.87 (0.78, 0.97)	0.002
Model 3	1.00	0.86 (0.79, 0.93)	0.86 (0.77, 0.96)	0.88 (0.79, 0.98)	0.006
Flavanones:					
Range of intake, mg	0.0	0.1 ^6^–0.5	0.6–11.8	>11.8	
RR (99% CI):					
Model 1	1.00	0.99 (0.91, 1.09)	0.93 (0.84, 1.03)	0.85 (0.76, 0.95)	<0.001
Model 2	1.00	1.01 (0.92, 1.11)	0.95 (0.87, 1.04)	0.88 (0.79, 0.97)	0.002
Model 3	1.00	1.01 (0.92, 1.11)	0.96 (0.88, 1.05)	0.89 (0.80, 0.98)	0.004
Flavones:					
Range of intake, mg	≤0.1	0.2–0.5	0.6–1.1	>1.1	
RR (99% CI):					
Model 1	1.00	0.96 (0.85, 1.07)	0.89 (0.80, 0.99)	0.91 (0.82, 1.01)	0.006
Model 2	1.00	0.97 (0.86, 1.09)	0.92 (0.83, 1.02)	0.96 (0.87, 1.06)	0.12
Model 3	1.00	0.98 (0.86, 1.11)	0.94 (0.85, 1.04)	0.99 (0.89, 1.10)	0.61
Flavonols:					
Range of intake, mg	≤6.3	6.4–13.3	13.4–25.7	>25.7	
RR (99% CI):					
Model 1	1.00	0.92 (0.84, 1.01)	0.86 (0.78 0.94)	0.92 (0.81, 1.05)	0.04
Model 2	1.00	0.94 (0.86, 1.03)	0.89 (0.82, 0.97)	0.97 (0.85, 1.09)	0.24
Model 3	1.00	0.95 (0.87, 1.04)	0.91 (0.83, 0.99)	0.99 (0.88, 1.11)	0.53

Abbreviations: RR, relative risk; high+ CVD risk, high, very high, or extremely high risk of cardiovascular disease; WWEIA, What We Eat in America; NHANES, National Health and Nutrition Examination Survey; CI, confidence interval. ^1^ RRs obtained from average marginal predictions (adjusted prevalence estimates) in the fitted logistic model [36,37]. Prevalence estimates were adjusted for the following: Model 1—energy, sex, and age (continuous); Model 2—all characteristics included in Model 1 plus race/ethnicity, income as percent of poverty, exercise status, smoking status, and health status; Model 3—all characteristics included in Model 2 plus dietary intakes of dietary fiber and added sugars. See Table 1 for explanations of characteristics. ^2^ Includes all those classified as being at high, very high, or extremely high risk of CVD on the basis of body mass index and waist circumference, as delineated in Reference [6]. ^3^ For total flavonoids, flavan-3-ols, flavones, and flavonols, intake categories are quartiles. Due to >25% of the population having zero intake of anthocyanidins (34%) and flavanones (35%), the first category for each of those classes was composed of all individuals with intake = 0.0, and those with intake >0.0 were divided into tertiles. ^4^ *p* value based on (first order) orthogonal polynomial contrasts of the adjusted prevalence estimates by category of flavonoid (total or class) intake. ^5^ In addition to the flavonoid classes listed in this table, total flavonoids also include isoflavones. ^6^ Includes non-zero intakes <0.05 mg.

**Table 5:** Prevalence and RR ^1^ of being classified at high+ CVD risk ^2^ by category ^3^ of flavonoid intake, males and females age ≥20 years, WWEIA, NHANES 2007–2010.

Prevalence, and RR by Flavonoid Class and Sex	Category of Flavonoid Intake				*p* for Trend ^4^
	1	2	3	4	
Total flavonoids ^5^:					
Male:					
Range of intake, mg	≤20.2	20.3–54.5	54.6–220.4	>220.4	
Prevalence, % (SE)	53.1 (1.4)	45.0 (2.6)	42.7 (2.5)	45.8 (2.0)	0.005
RR (99% CI)	1.00	0.85 (0.73, 0.98)	0.80 (0.67, 0.96)	0.86 (0.75, 0.99)	
Female:					
Range of intake, mg	≤17.7	17.8–51.6	51.7–240.5	>240.5	
Prevalence, % (SE)	61.8 (1.9)	60.1 (2.0)	56.4 (1.65)	58.1 (1.8)	0.12
RR (99% CI)	1.00	0.97 (0.89, 1.06)	0.91 (0.82, 1.02)	0.94 (0.82, 1.07)	
Anthocyanidins:					
Male:					
Range of intake, mg	0.0	0.1 ^6^–1.7	1.8–7.9	>7.9	
Prevalence, % (SE)	49.4 (1.5)	50.3 (2.2)	43.2 (2.0)	41.8 (2.3)	<0.001
RR (99% CI) ^6^	1.00	1.02 (0.91, 1.14)	0.87 (0.77, 1.00)	0.85 (0.71, 1.01)	
Female:					
Range of intake, mg	0.0	0.1 ^6^–1.9	2.0–12.7	>12.7	
Prevalence, % (SE)	63.3 (1.9)	55.6 (2.5)	62.9 (1.8)	53.2 (1.6)	0.002
RR (99% CI)	1.00	0.88 (0.75, 1.03)	0.99 (0.89, 1.11)	0.84 (0.76, 0.93)	
Flavan-3-ols:					
Male:					
Range of intake, mg	≤3.7	3.8–12.9	13.0–65.5	>65.5	
Prevalence, % (SE)	55.1 (1.8)	42.0 (2.3)	43.7 (2.4)	45.8 (1.9)	0.005
RR (99% CI)	1.00	0.76 (0.65, 0.89)	0.79 (0.66, 0.96)	0.83 (0.72, 0.96)	
Female:					
Range of intake, mg	≤2.9	3.0–12.4	12.5–164.9	>164.9	
Prevalence, % (SE)	62.1 (2.0)	58.7 (2.0)	57.3 (1.7)	58.2 (1.9)	0.17
RR (99% CI)	1.00	0.95 (0.85, 1.05)	0.92 (0.83, 1.02)	0.94 (0.82, 1.07)	
Flavanones:					
Male:					
Range of intake, mg	0.0	0.1 ^6^–0.5	0.6–14.5	>14.5	
Prevalence, % (SE)	47.4 (1.7)	49.0 (2.3)	46.6 (2.4)	42.9 (2.2)	0.06
RR (99% CI)	1.00	1.03 (0.90, 1.19)	0.98 (0.87, 1.12)	0.91 (0.78, 1.06)	
Female:					
Range of intake, mg	0.0	0.1 ^6^–0.5	0.6–9.3	>9.3	
Prevalence, % (SE)	61.4 (1.5)	62.4 (2.6)	56.5 (1.9)	54.9 (2.3)	0.01
RR (99% CI)	1.00	1.02 (0.91, 1.13)	0.92 (0.82, 1.03)	0.89 (0.78, 1.02)	
Flavones:					
Male:					
Range of intake, mg	≤0.1	0.2–0.5	0.6–1.2	>1.2	
Prevalence, % (SE)	48.5 (2.1)	46.4 (2.2)	44.4 (2.0)	47.2 (2.2)	0.43
RR (99% CI)	1.00	0.96 (0.80, 1.14)	0.92 (0.80, 1.05)	0.97 (0.86, 1.10)	
Female:					
Range of intake, mg	≤0.1	0.2–0.4	0.5–1.0	>1.0	
Prevalence, % (SE)	60.0 (2.5)	58.1 (2.0)	57.5 (1.9)	60.6 (1.5)	0.89
RR (99% CI)	1.00	0.97 (0.84, 1.12)	0.96 (0.83, 1.11)	1.01 (0.89, 1.14)	
Flavonols:					
Male:					
Range of intake, mg	≤7.4	7.5–15.2	15.3–29.2	>29.2	
Prevalence, % (SE)	48.1 (1.9)	44.1 (2.2)	45.9 (1.7)	48.3 (2.4)	0.78
RR (99% CI)	1.00	0.92 (0.79, 1.06)	0.95 (0.84, 1.08)	1.00 (0.85, 1.19)	
Female:					
Range of intake mg	≤5.4	5.5–11.7	11.8–23.2	>23.2	
Prevalence, % (SE)	59.8 (2.2)	58.7 (1.7)	58.0 (2.1)	59.8 (2.3)	0.93
RR (99% CI)	1.00	0.98 (0.87, 1.10)	0.97 (0.85, 1.10)	1.00 (0.86, 1.16)	

Abbreviations: RR, relative risk; high+ CVD risk, high, very high, or extremely high risk of cardiovascular disease; WWEIA, What We Eat in America; NHANES, National Health and Nutrition Examination Survey; SE, standard error; CI, confidence interval. ^1^ RRs obtained from average marginal predictions (adjusted prevalence estimates) in the fitted logistic model [36,37]. Prevalence estimates were adjusted for energy, age (continuous), race/ethnicity, income as percent of poverty, exercise status, smoking status, health status, and dietary intakes of dietary fiber and added sugars. See Table 1 for explanations of characteristics. ^2^ Includes all those classified as being at high, very high, or extremely high risk of CVD on the basis of body mass index and waist circumference, as delineated in Reference [6]. ^3^ For total flavonoids, flavan-3-ols, flavones, and flavonols, intake categories are quartiles. Due to >25% of the population having zero intake of anthocyanidins (36% of males and 31% of females) and flavanones (38% of males and 33% of females), the first category for each of those classes was composed of all those with intake = 0.0, and those with intake >0.0 were divided into tertiles. ^4^ *p* value based on (first order) orthogonal polynomial contrasts of the adjusted prevalence estimates by category of flavonoid (total or class) intake. ^5^ In addition to the flavonoid classes listed in this table, total flavonoids also include isoflavones. ^6^ Includes non-zero intakes <0.05 mg.

## EEO/Non-Discrimination Statement

USDA is an equal opportunity provider and employer.
